# The Critical Roles and Mechanisms of Immune Cell Death in Sepsis

**DOI:** 10.3389/fimmu.2020.01918

**Published:** 2020-08-25

**Authors:** Zhenxing Cheng, Simon T. Abrams, Julien Toh, Susan Siyu Wang, Zhi Wang, Qian Yu, Weiping Yu, Cheng-Hock Toh, Guozheng Wang

**Affiliations:** ^1^Department of Clinical Infection, Microbiology and Immunology, Institute of Infection and Global Health, University of Liverpool, Liverpool, United Kingdom; ^2^Medical School, Southeast University, Nanjing, China; ^3^Wirral University Teaching Hospitals NHS Foundation Trust, Wirral, United Kingdom; ^4^Royal London Hospital, London, United Kingdom; ^5^Liverpool University Hospitals NHS Foundation Trust, Liverpool, United Kingdom

**Keywords:** sepsis, extensive immune cell death, damage-associated molecular patterns (DAMPs), multiple organ dysfunction syndrome (MODS), extracellular histones, immunosuppression

## Abstract

Sepsis was first described by the ancient Greek physicians over 2000 years ago. The pathophysiology of the disease, however, is still not fully understood and hence the mortality rate is still unacceptably high due to lack of specific therapies. In the last decade, great progress has been made by shifting the focus of research from systemic inflammatory response syndrome (SIRS) to multiple organ dysfunction syndrome (MODS). Sepsis has been re-defined as infection-induced MODS in 2016. How infection leads to MODS is not clear, but what mediates MODS becomes the major topic in understanding the molecular mechanisms and developing specific therapies. Recently, the mechanism of infection-induced extensive immune cell death which releases a large quantity of damage-associated molecular patterns (DAMPs) and their roles in the development of MODS as well as immunosuppression during sepsis have attracted much attention. Growing evidence supports the hypothesis that DAMPs, including high-mobility group box 1 protein (HMGB1), cell-free DNA (cfDNA) and histones as well as neutrophil extracellular traps (NETs), may directly or indirectly contribute significantly to the development of MODS. Here, we provide an overview of the mechanisms and consequences of infection-induced extensive immune cell death during the development of sepsis. We also propose a pivotal pathway from a local infection to eventual sepsis and a potential combined therapeutic strategy for targeting sepsis.

## Sepsis

Sepsis is still the leading cause of death in most intensive care units (ICU) with an unacceptably high mortality rate (10–20%), although there has been a significant decrease in mortality rates in recent decades (from 1994 to 2014) (1, 2). Center for Disease Control in the United States estimated that over half a million people developed sepsis there per year with about a 1.5% increase per annum (3). A recent investigation of a cohort of 568 patients who died in six hospitals in the United States showed that sepsis presented in 300 patients (52.8%) and was the most common immediate cause of death in 198 patients (34.9%), indicating that sepsis is still the major cause of death in hospitals (4). For years, it was believed that high morbidity and mortality were due to systemic inflammatory response syndrome (SIRS), but many clinical trials to inhibit inflammation failed to improve survival (5–7). In 2016, sepsis has been redefined as multiple organ dysfunction syndrome (MODS) caused by a dysregulated host response to infection (8) and is now termed Sepsis-3. This has changed the focus from SIRS (9, 10) to MODS. Thus, finding what mediates MODS is now the major challenge in understanding the pathophysiology of sepsis (11).

## Discovery of Immune Cell Death in Sepsis

Cheadle et al. (12) reported that a significant lymphopenia occurred in a group of trauma patients with sepsis. Years later, lymphopenia in sepsis began to attract increased attention (13–16). In human, depletion of both B cells and CD4+ T lymphocytes caused by sepsis-induced apoptosis were reported (16). In baboon and murine sepsis models, extensive apoptosis of lymphoid tissue was also found (17–19). Rapidly progressing lymphocyte exhaustion after severe sepsis has been widely recognized (20) and early circulating lymphocyte apoptosis was associated with poor outcome in patients with sepsis (21, 22). Thus, a number of research groups have focused on the role of altered cell death in contributing to MODS in sepsis and clinical trials for a new type of therapy has emerged (23–26).

## Types of Immune Cell Death and Clinical Relevance

Lymphocyte death occurs in the spleen, thymus, and lymphoid tissues (27). The peripheral lymphocyte count is also dramatically reduced in both sepsis models and patients (16, 22, 24). Changes in the subsets of lymphocyte involved varies depending on the bacterial origin of sepsis (28), but there is no doubt that both T and B lymphocyte subsets are significantly changed by sepsis. CD3+, CD3+CD4+, and CD3+CD8+ lymphocyte counts drop significantly in septic patients, while CD3+CD4+ lymphocytes return to normal after 14 days in most patient survivors, but this is not true of the CD3+CD8+ counts (29). The ratio of Th1/Th2 helper cells has been found to be significantly lower in sepsis (30). Circulatory Th1, Th2, Th17, and Treg as well as Th1/CD4 ^+^ ratios are significantly lower in non-survivors compared to survivors (31). The αβ and γδ T cell subsets are all reduced in sepsis, but the CD3^+^ CD56^+^ γδ T cells show the largest decrease, and their loss is strongly associated with septic severity and mortality (32, 33). Sepsis causes progressive and profound depletion of B lymphocytes in patients (16). Thus, the percentage of CD19+CD23+ was significantly lower in patients who died of septic shock than in survivors (34). In a mouse poly-microbial sepsis model, substantial apoptosis of lamina propria B cells mediated by FasL has been reported (35).

Not only are B and T lymphocytes **s**usceptible to programmed cell death, many other types of immune cells including neutrophils, macrophages and dendritic cells are also vulnerable to cell death in sepsis (22, 36, 37). Neutrophils are the first line of defense against invading bacteria. Neutrophils phagocytose bacteria or form neutrophil extracellular traps (NETs), and both these mechanisms are critical for clearance of invading bacteria (38). After taking up bacteria, neutrophils undergo a respiratory burst and die (39). NETs formation is also a novel program for cell death (40–42). Therefore, large numbers of neutrophils die during sepsis. In mouse models, apoptosis of mouse peritoneal macrophages may be due to the release of HMGB1 in sepsis (43). Dendritic cells have unique capabilities to regulate the activity and survival of T and B cells. Thus splenic interdigitating dendritic cells (IDCs) and follicular dendritic cells (FDCs) initially expand in sepsis. The FDCs expand to fill the entire lymphoid zone of spleen, which is otherwise occupied by B cells (44). Twelve hours after the onset, these dendritic cells undergo apoptosis (44). In contrast, natural killer (NK) cell counts increase in early sepsis and higher levels predict mortality in severe sepsis (45). Thus, the ratio of NK cells to CD4+ lymphocytes was used to predict the mortality of patients with sepsis (46). NK cells also contribute to the lethality of a murine model of sepsis, and NK cell-depleted and NK cell-deficient mice showed much high survival rates than wild type controls (47).

## Mechanisms of Immune Cell Death

Apoptosis is the major mechanism of lymphocyte death in sepsis (35, 48). Both the death receptor and mitochondrial pathways activated by multiple triggers are involved in apoptosis of a broad range of subsets of lymphocyte (49). Apoptosis could occur via p53-dependent and -independent pathways (50). The increase in apoptosis in the thymus, spleen, lungs, and gut during poly-microbial sepsis of mice is mediated by FasL via death receptors, but not by endotoxins nor TNF-α (14, 35). Monocytes can induce Fas-mediated apoptosis of T lymphocytes (51). Caspase-1 is involved in apoptosis of splenic B lymphocytes (52). Activation of caspase-3 and externalization of phosphatidylserine in CD4+, CD8+, and CD19+ lymphocytes were reported in patients with sepsis (53). Activation of programmed cell death ligand 1 (PD-L1) pathway is involved in T cell exhaustion in patients with sepsis (54). In addition, endoplasmic reticulum (ER) stress can mediate lymphocyte apoptosis in sepsis (55). Bcl-2 is an anti-apoptosis protein and is found to be reduced in sepsis (56). Overexpression of Bcl-2 in septic mice provides protection by decreasing lymphocyte apoptosis (57, 58). In CD4^+^ T and B lymphocytes isolated from septic patients, the Bcl-2 protein was decreased but the expression of pro-apoptotic proteins Bim, Bid, and Bak were massively upregulated (23, 53). It has also been reported that overexpression of histamine H4 receptors counteracts the effect of NF-kB in contributing to splenic cell apoptosis in sepsis (59).

There is no doubt that multiple factors are involved in lymphocyte apoptosis, but the detailed molecular mechanisms are still not fully understood. In addition, apoptosis has been the major focus of cell death in last two decades, but recently other processes have emerged, e.g., pyroptosis, necroptosis, ferroptosis, parthanatos, entotic cell death, NETotic cell death, immunogenic cell death, and mitotic catastrophe, to explain the complexity of cell death (60). Pyroptosis is caused by rapid plasma-membrane rupture by non-selective gasdermin-D pore and releases of DAMPs (61). Neutrophil and endothelial cell pyroptosis has been considered as a major pathological factor in sepsis (62, 63). Increased membrane permeabilization in necroptosis releases specific DAMPs, and lipid peroxidation in ferroptosis may be involved in renal failure (64–66). These regulated cell deaths may turn to necrosis if their resolution is delayed (67). The roles and mechanisms of different types of cell death in sepsis is far from clear and more work need to be done to understand how the immune cells die so extensively in sepsis.

Neutrophil respiratory burst and NETosis all involve generation of reactive oxygen species (ROS) and NADPH oxidase pays a critical role (40). Endotoxin reduced CD95-mediated neutrophil apoptosis occurs via cIAP-2 activation and the degradation of caspase-3 (68). The detailed molecular mechanisms of neutrophil respiratory burst, NETosis, and homeostasis will not be discussed in this review.

In summary, the types of cell death and underlying molecular mechanisms are still not fully understood, although the subpopulations of immune cells that die during sepsis is almost clarified.

## Roles and Consequence of Immune Cell Death

It is known that the extent of immune cell death is strongly associated with severity and mortality of sepsis. However, the biological roles are still not clear. The direct cause-effect relationship of extensive immune cell death with sepsis has not yet been proven. When splenectomy to remove the largest lymph organ in mice prior to septic modeling was undertaken, it is found that this procedure protects mice against secondary sepsis (69, 70). This observation suggests that extensive splenocyte death is potentially pathogenic in sepsis. Neutrophil death, particularly NETosis, has been reported to be involved in the development of multiple organ failure in sepsis (71–73). Abrams et al. (74) recently showed that strong NETs formation mainly occurs in severe sepsis and is associated with disseminated intravascular coagulation (DIC) and ultimately poor outcomes. Patel et al. (75) recently showed that a reduction in *ex vivo* PMA-induced NETosis of neutrophils isolated from patients with severe sepsis is associated with poorer outcomes. This observation demonstrates the pathological role of *in vivo* NETs formation, a mechanism that eliminates the majority of pro-NETosis neutrophils. This result is also consistent with the current general consensus (72, 74). However, the pathological role of immune cell death in sepsis is still not fully understood, but the following mechanisms are widely considered to be very important.

### DAMPs and Histone Release

The “danger” theory was proposed by Matzinger in 1994 (76) that damaged cells initiate immune responses by releasing substances were termed damage-associated molecular patterns (DAMPs) by Walter Land in 2003 (77). DAMPs represent danger-associated or damage-associated molecular patterns, which are released from the cell through activation of inflammasome or passively following cell death (78–80) and recognized by pattern recognition receptors (PRRs), including Toll-like receptors (TLRs) NOD-like receptors, DNA sensors, C-type lectin receptor, and non-PRR DAMP receptors, including RAGE receptor (81). Many DAMPs that origin from extracellular matrix and different components or organelles of cells have been identified, such as histones, DNAs, HMGB1, heat shock protein, and ATP. More information can be found in a recent review (82). In sepsis, a large number of immune cell death releases a large quantity of DAMPs (83, 84). Similarly, NETs are released from neutrophils during inflammation (41). These NETs are brokendown into free DNA and histones and become a source of DAMPs (72, 85). DAMPs trigger the host's immune response, activate coagulation and mediate MODS (86–88). Therefore, they play a central role in development of sepsis and its progression (84, 89). DAMPs include a large group of molecules and are involved in different pathological processes during sepsis.

Release of chromatin protein HMGB1 triggers inflammation and mediates endotoxin lethality in mice (90, 91). HMGB1 facilitates LPS entering cells to trigger pyroptosis, which plays an important role in sepsis (63, 92, 93). In 2009, extracellular histones were shown to be major mediators of death in sepsis (94) and have attracted more and more attention. Extracellular histones bind to the cell membrane and form pores to allow calcium influx which leads to calcium overload, which directly damages cells that contacted (87, 88). Histones also induce rapid thrombocytopenia, increase thrombin generation and contribute to DIC (95–99). Anti-histone antibodies and non-anticoagulant heparin neutralize extracellular histones and improves survival in sepsis (87, 88, 99–101). Recently, the role of extracellular histones in the development of MODS in critical illnesses and animal models, including sepsis, pancreatitis, and trauma, has been demonstrated (86). Mitochondrial DNA released into the cytosol or outside cells also serves as DAMPs and play important roles in sepsis (11, 102). In addition, circulating cell-free DNA is associated with poor outcomes in patients with severe sepsis (103–106). The pathological roles of these cell-free DNAs are not clear but strengthening blood clots resistant to fibrin lysis may facilitate DIC development (107). A recent report shows increased S100 proteins, including A8/A9 and A12, which are types of DAMPs, are associated with a higher risk of death in patients with sepsis (108).

### NETs Formation

Although NETs are an important source of DAMPs, NETs formation has specific roles in thrombosis, DIC and microcirculatory impairment. NETs formation induces organ injury and exacerbates the severity of sepsis (42, 73, 74, 109–112). Suppression of NETosis using PAD4 inhibitors or cleavage of NETs using DNase 1 improves survival in a murine sepsis model (113), but other reports showed the opposite effect (114, 115). Recently it has been reported that delayed, not early treatment with DNase 1 reduces organ injury and improves outcome in sepsis model (116). These observations strongly indicate the complex roles of NETs formation during sepsis.

### Coagulopathy and DIC

Sepsis-induced coagulopathy and DIC play a major role in microcirculatory impairment and MODS development (117). DAMPs play important roles in septic coagulopathy (118). Extracellular histones are the most important DAMPs that promote coagulation activation by inducing rapid thrombocytopenia, enhancing thrombin generation, impairing thrombomodulin-dependent protein C activation, damaging endothelial cells and increasing tissue factor activity (95–99). cfDNA exert both pro- and anti-fibrinolytic effects and NETs serve as scaffolds for immunothrombosis and promote intracellular coagulation together with platelets (107, 119, 120). The overall consequence is the development of coagulopathy and DIC, which significantly enhance disease severity and worsen the outcomes (74, 86, 99, 104, 105).

### Immune Suppression

As our understanding of the pathophysiology in sepsis has improved, we now know that the role of immunosuppression is more important than previously thought. IL-7, as an immune-adjuvant therapy that increased absolute lymphocyte counts and in circulating CD4+ and CD8+ T cells (3–4 fold), and T cell proliferation and activation (121), supports this contention. However, why IL-7 protected mice with sepsis but showed no effects on 28-days survival of patients with sepsis is not clear and further investigation is required (122). IL-15 is also reported to prevent apoptosis, reverse innate and adaptive immune dysfunction, and improve survival in murine models of sepsis (123). Changes associated with immunosuppression is more obvious in patients who died of sepsis than those who survived (31, 124). Immune cell death, particularly T and B lymphocytic apoptosis, is a major contributor to the development of immunosuppression (15, 32, 125), besides the usual anti-inflammatory cytokine release, such as that of IL-10 (126). Myeloid-derived suppressor cells (MDSCs) are closely related to neutrophils and monocytes. They are immature myeloid cells that have immunosuppressive functions and play important roles in the development of immunosuppression in sepsis (2, 127–129). DAMPs activate TLR-4 to enhance MDSCs accumulation (130). Many DAMPs possess both pro- and anti-inflammatory properties to induce both immune response and immunosuppression, which has been well-studied in trauma (131). Recently, the roles of PD-1 and PD-L1 in sepsis as key mediators of T-cell exhaustion in infections have been investigated (132, 133). Blocking PD-1 or PD-L1 inhibits lymphocytic apoptosis, reverses monocyte and immune dysfunction, and improves survival during sepsis (54, 134–136). Monneret et al. (137) demonstrated that after septic shock anti-inflammatory response became dominate with high IL-10 and low HLA-DR on monocytes, a surrogate marker of monocyte non-responsiveness (138). IL-7 and anti-PD-1 or blocking IL-10 reverse sepsis-induced immunosuppression, including increasing HLA-DR expression and IFN-γ production, and improve survival in mouse models (126, 139). Monitoring HLA-DR, PD-1, or PD-L1 may guide clinical immunotherapies (140). All available evidences showed no doubt that immunosuppression is the major pathological feature and immunotherapies will become a critical management in severe sepsis with poor outcomes.

In summary, the major consequence of immune cell death is the DAMPs release and NETs formation, both of which contribute to the development of coagulopathy and MODS. Another major consequence is immunosuppression. All these consequences are the major pathological changes during severe sepsis, strongly indicating that DAMPs and NETs are critical in the development of severe sepsis.

## Inhibition of Immune Cell Death in Sepsis and Potential Downstream Therapy

Caspase inhibitors, which inhibit apoptosis and improve the survival of immune cells, have been demonstrated to improve survival in sepsis. Thus, caspase-3^−/−^ mice have decreased levels of apoptosis (141, 142). Increasing anti-apoptotic proteins, such as Bcl-2, or decreasing pro-apoptotic proteins, such as Bim, reduces immune cell apoptosis and improves survival in septic animal models (57, 58, 142–145). The Protease Inhibitor (PI) class of antiretroviral agents also significantly improved survival of mouse septic models by reducing lymphocyte apoptosis (146). These anti-apoptosis therapies have been demonstrated in animal models (147), but there have been no successful clinical trials in humans as yet.

Therapies with immune modulators have attracted more attention in recent years. The success of the IL-7 clinical trial shed some light on the management of sepsis (121). Immunotherapy is potentially a major strategy (145, 148, 149), but the focus of research has shifted from simply suppressing the immune response to immune modulation and precision medicine based on immune status (148, 150–153). Targeting immune cell checkpoints during sepsis is also a potential therapeutic strategy (154).

Another promising strategy is to neutralize DAMPs, including histones, DNAs and HMGB1. Anti-histone therapy has been proposed by Xu et al. from 2009 (94). Anti-histone antibodies or heparin can neutralize extracellular histones and reduce their toxicity so as to increase survival rates in septic animal models, but no clinical trial has been reported yet (86–88, 94, 100). Normal heparin has anticoagulant activity which may cause side effects if it is used at a wrong time with high doses. Non-anticoagulant heparin has been developed and hold the promise for future clinical application (100, 155). DNase 1, used to digest free DNA or NETs, has also been shown to increase the survival rate of septic animal models (116, 156). Many reagents targeting HMGB1, its release or downstream pathways have been reported, but no drug has yet been fully developed for clinical management of sepsis (157, 158).

Correction of downstream events, such as coagulopathy, have been trialed. Activated protein C, an anti-coagulant enzyme, was used clinically for a few years, but was withdrawn from the market due to failure in randomized controlled trials (159). It is very difficult to justify the correct time to use anti-coagulants and fibrinolysis reagents, such as low-molecular-weight heparin, antithrombin, thrombomodulin, and tissue factor inhibitors (117). Therefore, anti-coagulant therapy for sepsis is difficult to use clinically. Developing therapies to target upstream events appears a better strategy.

## Clinical Perspective

**Figure 1:**
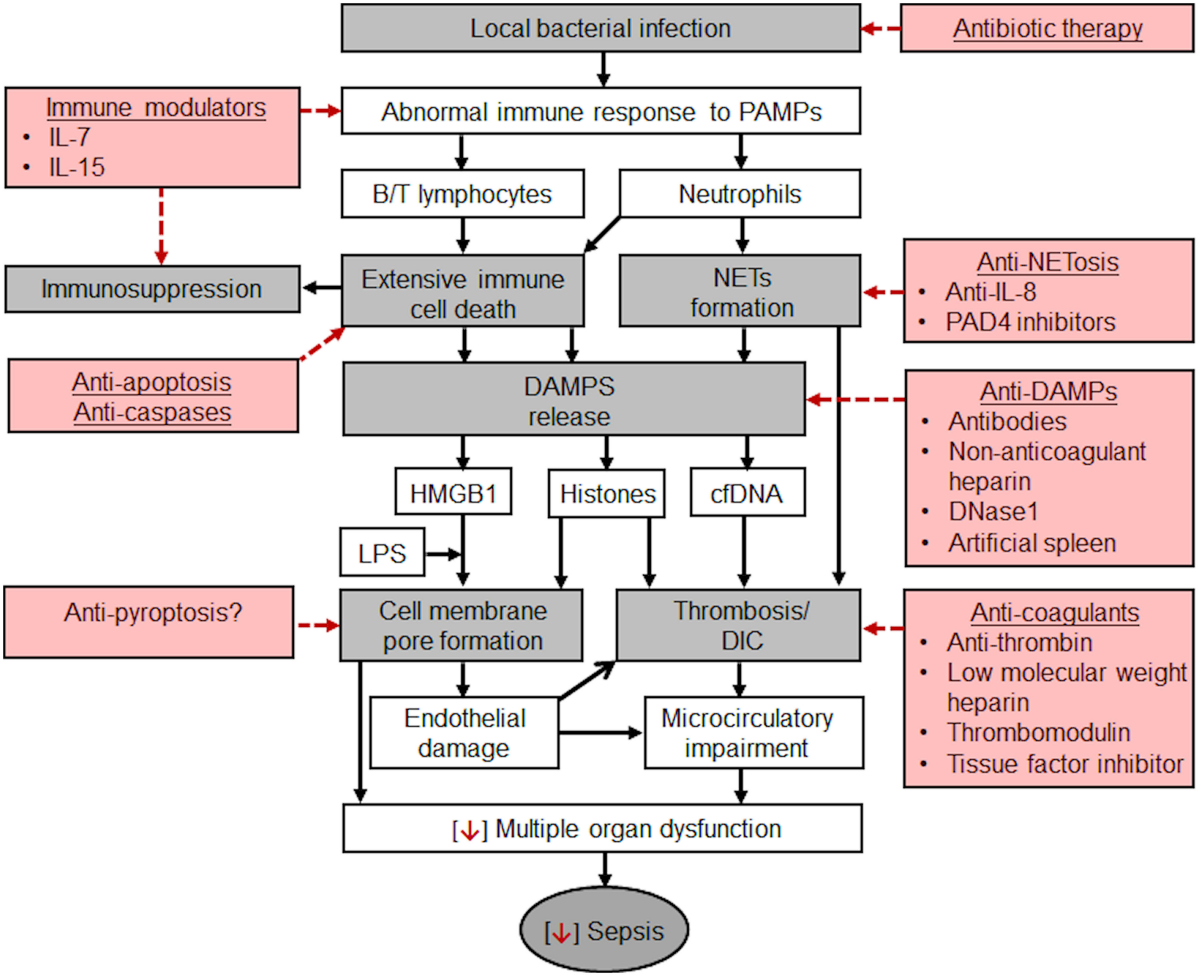
Potential pathological mechanisms of sepsis which develops from a local bacterial infection and potential therapeutic strategies. Gray boxes: pathways from a local infection to sepsis. Once a local bacterial infection causes host abnormal immune responses to pathogen-associated molecular pattern (PAMPs), extensive immune cell death, including B/T lymphocytes (spleen, thymus, lymphoid tissues, and peripheral blood), and neutrophils could occur and result in immunosuppression. Neutrophils could also form NETs. NETs and immune cell death could release a large quantity of DAMPs, mainly HMGB1, cfDNA, and histones. HMGB1 can delivers LPS into cells to trigger pyroptosis by forming pores in the cell membrane. Extracellular histones could also bind to cell membrane to form pores which may cause calcium overload and subsequently endothelial damage and organ injury. Indirectly, extracellular histones activate coagulation to form thrombi in the microvascular lumen to impair microcirculation. cfDNA could serve as scaffolds for thrombosis or stabilize thrombi by increasing their resistance to fibrinolysis. Microcirculatory impairment is the major feature of sepsis and a major contributor to MODS. Red boxes: Potential therapeutic strategies. Besides early diagnosis, prompt use of effective antibiotics, and supportive therapies, such as maintaining blood pressure and circulation, improving microcirculation, and protecting individual organs, the potential specific therapies include the combination of modulating immune status, preventing immune cell death and NETosis, neutralizing or clearing DAMPs. These new approaches could become the leading research directions in reducing the mortality rate of sepsis.

Sepsis was first described by the ancient Greek physicians. Despite millennia of experience with this illness, we are still investigating the nature of sepsis. In the last decade, great progress has been made by shifting the focus of research from SIRS to MODS. However, the pathophysiology of sepsis is still not fully understood, particularly the roles of extensive immune cell death and DAMPs. Many types of DAMPs could directly or indirectly mediate MODS by their cytotoxicity or by triggering inflammation and activating coagulation, respectively. Therefore, the axis of infection, immune response, immune cell death, DAMPs release and MODS could be the central pathological pathway in the transition of a local infection to sepsis (Figure 1).

Targeting this central pathological pathway is already underway. However, fully understand the pathophysiology of sepsis is still the first task toward the success in clinical management.

## Discussion

There is no doubt that extensive immune cell death is a major driver of sepsis. This mainly involves T and B lymphocyte apoptosis in the spleen, thymus, lymphoid tissues, and circulation. Neutrophil apoptosis, respiratory burst, and NETosis are also involved in this event. Macrophages and dendritic cells may also be involved, but their contributions may be negligible. However, the mechanism of how bacterial infection leads to extensive immune cell death is still not fully understood. Moreover, significant gaps still exist in our understanding of how extensive immune cell death proceeds to the development of sepsis. The obvious consequence of immune cell death would be immunosuppression but no direct link has been demonstrated. It is clear that the release of large quantities of DAMPs can enhance inflammation, directly damage endothelial cells, impair microcirculation and cause multiple organ injury, but to what extent these DAMPs contribute to the development of sepsis is still unclear. Some DAMPs, such as histones and NETs, strongly activate coagulation and eventually lead to DIC. Therefore, the importance of DAMPs in sepsis development and progression cannot be underestimated. In the future, targeting the axis of immune cell death-DAMPs release-and microcirculatory impairment, will become themost comprehensive strategy to reduce the unacceptably high mortality rate of sepsis.

## Author Contributions

ZC, ZW, and QY wrote the first draft. JT and SW edited the reference. SA drew the diagram. WY, C-HT, and GW supervised the work and edited final version of paper. All authors contributed to the article and approved the submitted version.

## Conflict of Interest

The authors declare that the research was conducted in the absence of any commercial or financial relationships that could be construed as a potential conflict of interest.
